# Human Cytomegalovirus Infection Enhances NF-κB/p65 Signaling in Inflammatory Breast Cancer Patients

**DOI:** 10.1371/journal.pone.0055755

**Published:** 2013-02-13

**Authors:** Mohamed El-Shinawi, Hossam Taha Mohamed, Eslam A. El-Ghonaimy, Marwa Tantawy, Amal Younis, Robert J. Schneider, Mona Mostafa Mohamed

**Affiliations:** 1 Department of General Surgery, Faculty of Medicine, Ain Shams University, Cairo, Egypt; 2 Department of Zoology, Faculty of Science, Cairo University, Giza, Egypt; 3 Department of Microbiology, School of Medicine, New York University, New York, New York, United States of America; University of Regensburg, Germany

## Abstract

Human Cytomegalovirus (HCMV) is an endemic herpes virus that re-emerges in cancer patients enhancing oncogenic potential. Recent studies have shown that HCMV infection is associated with certain types of cancer morbidity such as glioblastoma. Although HCMV has been detected in breast cancer tissues, its role, if any, in the etiology of specific forms of breast cancer has not been investigated. In the present study we investigated the presence of HCMV infection in inflammatory breast cancer (IBC), a rapidly progressing form of breast cancer characterized by specific molecular signature. We screened for anti-CMV IgG antibodies in peripheral blood of 49 non-IBC invasive ductal carcinoma (IDC) and 28 IBC patients. In addition, we screened for HCMV-DNA in postsurgical cancer and non-cancer breast tissues of non-IBC and IBC patients. We also tested whether HCMV infection can modulate the expression and activation of transcriptional factor NF-κB/p65, a hallmark of IBC. Our results reveal that IBC patients are characterized by a statistically significant increase in HCMV IgG antibody titers compared to non-IBC patients. HCMV-DNA was significantly detected in cancer tissues than in the adjacent non-carcinoma tissues of IBC and IDC, and IBC cancer tissues were significantly more infected with HCMV-DNA compared to IDC. Further, HCMV sequence analysis detected different HCMV strains in IBC patients tissues, but not in the IDC specimens. Moreover, HCMV-infected IBC cancer tissues were found to be enhanced in NF-κB/p65 signaling compared to non-IBC patients. The present results demonstrated a correlation between HCMV infection and IBC. Etiology and causality of HCMV infection with IBC now needs to be rigorously examined.

## Introduction

Recent studies suggested that chronic viral infection may have a role in cancer etiology including breast cancer [1]. DNA tumor viruses such as Epstein–Barr Virus (EBV) and HCMV are of particular interest has been suggested to be involved in certain human cancers given the fact that they are often endemic in the human population [2]. EBV has been established as a causal agent in undifferentiated nasopharyngeal carcinoma and Burkitt’s lymphoma [3], and was reported to be present at high incidence in breast carcinoma of Egyptian [4] and Tunisian [5] women. HCMV genes and proteins have been detected in different types of human cancers [6], including colorectal cancer [7], prostate cancer [8], breast cancer [9], [10], mucoepidermoid carcinoma of salivary glands [11], glioblastomas [12], [13] and medulloblastomas [14]. Studies showed that medulloblastoma and glioma cancer cells possess a suitable environment for HCMV virus to exert its “oncogenic potential” [15], [16]. Notably, HCMV infection induces the secretion of inflammatory cytokines and growth factors that also promote carcinogenesis [6].

The ability of HCMV to infect a wide variety of cells that constitute the tumor microenvironment, including monocytes/macrophages, fibroblasts and endothelial cells, raises the possibility that instead of a directly transforming role, HCMV might be strongly associated with certain human cancers through a viral “oncomodulatory” role in which the virus regulates immune cell function and reprogramming cells towards greater tumor progression. For instance, infection of monocytes with HCMV stimulates differentiation to macrophages that possess properties of both M1 (pro-inflammatory) and M2 (tumor promoting) phenotypes, including the secretion of cytokines and chemokines that favor immune evasion and cancer progression [17]. An increase in the secretion of tumor promoting, M2-type cytokines was found following HCMV virus infection, resulting from activation of the NF-kappa-B (NF-κB) transcription factor and the phosphoinositol-3-kinase (PI3K) signaling pathway [18]. Similarly, HCMV infects tumor associated fibroblasts [7] and dermal fibroblasts, resulting in stimulation of the NF-κB signaling pathway and secretion of inflammatory cytokines [19]. Endothelial cells infected with HCMV secrete cytokines and chemokines including interleukin-6 (IL-6), granulocyte-macrophage colony-stimulating factor (GM-CSF), macrophage inflammatory protein-1 (MIP-1), monocyte chemotactic protein-1 (MCP-1) and matrix metalloproteinases (MMPs) that promote carcinogenesis [20]. Recent studies conducted by Fiorentini and colleagues showed that HCMV also has a significant ability to infect lymphatic endothelial cells (LECs), also altering their secretion of cytokines compared to those secreted by non-infected LECs. HCMV infected LECs were found to produce a panel of cytokines and chemokines (secretome) that induces migration and tubule formation which underlie the angiogenic properties of endothelial cells, even when cultured *in-vitro* on basement membrane extracts. The mechanism of tubule formation was found to be modulated by strongly expressed cytokines such as IL-6 and GM-CSF, as well as by MMPs, all detected in the secretome of HCMV-infected LECs [21]. Furthermore, HCMV infection can inhibit apoptosis, promote cell survival, increase cell proliferation and drug resistance, in part by induction of the p53 tumor suppressor gene and through other poorly established mechanisms [22]. Interestingly, medulloblastoma tumors and cell lines were found to express US28, an HCMV encoded chemokine receptor that promotes neoplastic transformation and cell migration towards RANTES and MCP-1 chemo-attractants [13].

IBC is a particularly aggressive and highly metastatic form of breast cancer characterized by very rapid onset of progression over a period of weeks to a few months, with (lymph) angiogenesis, a pronounced ability to invade the dermal lymphatics, formation of lymphatic tumor emboli and aggressively infiltrating lymph nodes [23]. Lymphatic tumor emboli characterize the unique phenotype of IBC and promote its spread and dissemination to different organs [23]. More than a fourth of all cases of IBC are pregnancy associated, occurring during or within two years of pregnancy. Moreover, IBC occurs at a higher frequency in women of Northern African and Egyptian descent [24], [25]. The etiology of IBC is unknown, despite suggestions in the literature ranging from pollution, environmental factors, viral infection and or lifestyle [26], [27]. Thus, little is known about the underlying cause of IBC, particularly its rapid and often explosive presentation.

Our previous results showed that the IBC tumor microenvironment possesses high proteolytic activity [28], [29]. The increase in proteolytic activity and the high level of M2 macrophages in IBC lesions might suggest an involvement of HCMV in the etiology and progression of IBC.

In the present study we screened for evidence of HCMV infection in breast carcinoma/non-carcinoma tissues of non-IBC and IBC breast cancer tissues and patient sera, as well as normal breast tissues obtained from mammoplastic (breast reduction) surgery. Since, HCMV infection strongly enhances NF-κB activity, we tested the level of expression and activation of NF-κB/p65 in infected and non-infected carcinoma tissues of IBC and non-IBC patients. We found that the majority of IBC carcinoma tissues were infected with HCMV-DNA and characterized by over expression and activation of the NF-κB/p65 signaling molecule.

## Materials and Methods

### Reagents

HCMV IgG Chemiluminescence detection kit was purchased from Diasorin (Liaison, Italy). GeneJET™ Genomic DNA purification kit, GeneJET™ Gel Extraction Kit and green Taq polymerase master mix, were purchased from Fermentas (Burlington, ON, Canada). DNA ladder and ProSeiv®Color Protein marker were purchased from Lonza (Rockland, ME, USA). Total NF-κB/p65 and Phospho-NF-κB/p65(Ser^267^) antibodies were purchased from Cell Signaling Technology, Inc. (Boston, MA, USA). Antibody diluent with background reducing components and Dako Cytomation EnVision+ Dual Link System-HRP (DAB+) kits were purchased from Dako (Carpinteria, CA, USA). Horseradish Peroxidase (HRP) labeled goat anti-rabbit secondary antibody and 3, 3′, 5, 5′-tetramethylbenzidine (TMB) membrane peroxidase substrate were purchased from Kirkegaard and Perry Laboratories (KPL) Inc. (Gaithersburg, MD, USA). Permount® was purchased from Fisher Scientific (Pittsburgh, PA, USA). Unless otherwise stated all other reagents were from Sigma (St. Louis, MO, USA).

### Patients and Samples

For patient recruitment, Institutional Review Board (IRB) approval was obtained from the ethics committee of Ain-Shams University. All patients signed a consent form before participating in the study. Patients were clinically and pathologically diagnosed as breast cancer patients prior to enrollment during the period of January 2010 to January 2012, from the breast clinics of Ain Shams University Hospitals, and the Faculty of Medicine of Ain Shams University, Cairo, Egypt. Patients were stratified as non-IBC and IBC subgroups. IBC was diagnosed according to the American Joint Committee on Cancer (AJCC) guidelines as a T4d designation where patients presented with diffuse erythema, edema of the breast, skin orange peel (pead’orange) and pathological diagnosis of skin biopsies that showed tumor emboli with dermal invasion, a hallmark of IBC as we described previously [29]. Non–IBC patients were diagnosed by clinical examination, ultrasound, mammography, and confirmed by biopsy (tru-cut).Patients testing positive with HCV or HBV infection, or autoimmune diseases, were excluded from this study. By applying these criteria,77 breast cancer patients were enrolled in the study, 49 of them diagnosed as non-IBC and 28 diagnosed as IBC.

### Serological Assay

Five ml of peripheral blood was collected from each patient and healthy volunteers in Ethylenediaminetetraacetic acid (EDTA) tubes (Greiner Bio-one) for serological diagnosis of HCMV IgG antibodies. Blood was collected and centrifuged at 1500 rpm for 10 min to isolate plasma for serological tests. IgG antibodies against HCMV were measured in plasma samples of cases and controls using the Chemiluminescence technique and a Liaison device. The kit calculates HCMV IgG antibody concentrations which are expressed as IU/ml. Samples with HCMV IgG concentrations equal or more than 0.6 IU/ml were considered positive.

### Nested PCR

DNA was extracted from fresh breast tissue samples obtained during modified radical mastectomy using the GeneJET™ Genomic DNA purification kit as described in the kit guidelines. DNA of HCMV was detected using a nested PCR procedure [30]. Two sets of primers were specific for the fourth exon of the HCMV Immediate Early (IE) gene described by Zhang et al [30]. The sequences of the primers were as follows: the external primers 1a: 5′-GGTCACTAGTGACGCTTGTATGATGA-3′ and 1b 5′-GATAGTCGCGGGTACAGGGGACTCT-3′; the internal primers 2a 5′-AAGTGAGTTCTGTCGGGTGCT-3′ and 2b 5′-GTGACACCAGAGAATCAGAGGA-3′. Briefly, the first round of PCR was carried out in a 25 µl total volume containing 1 µl of each external upstream and downstream primers (10 pmol\µl), 3 µl of the DNA extract from fresh breast cancer tissue (and used as a template), 7.5 µl of free RNase water and 12.5 µl of Green Taq-polymerase master mix. Reaction conditions included an initial denaturation step of 94°C for 5 min, followed by 30 cycles of 94°C for 45 sec, 55°C for 45 sec, and 72°C for 45 sec, followed by terminal extension at 72°C for 10 min. The second round of PCR was performed in the same manner as the first, but using 3 µl of the first reaction as template, 1 µl of each internal upstream and downstream primers (10 pmol\µl) and an annealing temperature of 50°C for 45 sec. After nested PCR amplification, 10 µl of PCR products were electrophoresed on 2% agarose gel stained with ethidium bromide, and photographed by the GBOX-F3 gel documentation system Syngene (Syngene, MD, USA). All PCR amplifications were conducted using sterilized tubes and tips in a biosafety hood that was not previously exposed to HCMV. Controls without breast tissues tested negative, affirming the lack of contamination.

### DNA Sequencing of Nested PCR Product

Twenty two nested PCR products (10 non-IBC, 10 IBC and 2 positive control samples) were randomly selected, purified from the agrose gel using GeneJET™ Gel Extraction Kit Purified PCR product were commercially sequenced at Macrogen, Inc. (Rockville, MD, USA). Single-pass sequencing was performed on each sample using the same internal upstream and downstream primers of the nested PCR. Analysis of the DNA sequence results and multiple alignments were performed using the Basic Local Alignment Search Tool (BLAST) database.

### Sodium Dodecyl Sulfate-polyacrylamide Gel Electrophoresis (SDS-PAGE) and Immunoblotting

Fresh breast cancer tissues obtained during surgery were ground in liquid nitrogen and homogenized in RIPA buffer [25 mM Tris HCL pH 7.6, 150 mM NaCl, 1% Triton X-100, 1% Sodium deoxycholate and 0.1% SDS]. Equal amounts of tissue lysates were loaded based on protein concentration and resolved under reducing conditions using 12% SDS-PAGE, followed by transfer to Polyvinylidene fluoride (PVDF) membrane (Millipore, USA.). The membranes were blocked for 2 hrs with 5% non-fat dry milk in TBS-0.5% Tween 20, followed by incubation with total NF-κB/p65 antibody and phospho-NF-κB/p65 (Ser^276^) antibody then washed and incubated with 1∶10000 diluted peroxidase-labeled goat anti-rabbit secondary antibody. After washing, the bands were visualized by the addition of TMB. Once the color appeared the reaction was stopped by immersing membrane in water. Visualized bands were analyzed by ImageJ (National Institutes of Health, Bethesda, MA, USA) software, that measures the density of each band using β-actin as a loading control.

### Immunohistochemistry (IHC)

Immunohistochemical staining was performed on 4 µm-thick paraffin tissue sections, in duplicate. Tissue sections were first deparaffinized with overnight incubation in xylene and rehydrated through graded concentrations of ethanol. For antigen retrieval, slides were warmed in antigen retrieval buffer in the microwave for 3 min at 95°C, followed by cooling at room temperature for 20 min and washing 3 times with PBS. Endogenous peroxidase activity was blocked for 5 min using Dako Dual Endogenous Enzyme Block. After blocking tissue sections were washed 3 times with PBS then incubated for 90 min. at room temperature with primary total NF-κB/p65 and phospho-NF-κB p65 (Ser^276^) antibodies diluted in Dako antibody diluent-reduce background staining. Following this the tissues were Then washed 3 times with PBS and incubated with labeled polymer HRP Rabbit/Mouse [EnVision+ Dual Link System-HRP (DAB+)] for 30 min. Staining was achieved by adding 100 µl of DAB+ chromogen diluted 1∶50 in substrate buffer [EnVision+ Dual Link System-HRP (DAB+)] for 10 min. Nuclei were counterstained with hematoxylin then rinsed in tab water, finalization was carried out through graded concentrations of ethanol and xylene before slides were mounted using Permount® for microscopic examination. Negative control slides were run in parallel with each marker where primary antibody is replaced by PBS. The level of expression of markers was scored according to both the intensity of staining and the proportion of positive staining of carcinoma cells within the entire slide as we described previously [29]. Briefly score “0” when no immunostaining was detectable; score “1”, when > 10% of cells showed moderate to marked intensity; score “2” when 10–50% of cells showed staining of moderate to marked intensity and score “3” when > 50% of cells showed staining of moderate to marked intensity.

### Statistical Analysis

Data are expressed as mean ± standard deviation (S.D.). Statistical differences or comparison between two groups were assessed by Student’s t-test and Fisher’s exact test using SPSS 16.0 software (SPSS).

## Results

### Clinical and Pathological Characterization of IBC Versus Non-IBC Patients

Clinical and pathological characterization of patients enrolled in the present study is described in Table 1. Ages (mean ± SD) of non-IBC patients ranged from 29–73 years old (53.3±11.3) while ages of IBC patients ranged from 29–65 years old (48.9±9.9). Ages of Egyptian breast cancer patients were similar to Asian patients and significantly younger by a decade than breast cancer patients of Western countries [31]. Measurements of the tumor size showed that 61% of non-IBC patients had a tumor size ≤ 4 cm, with 34% having a tumor > 4 cm, while, 21% of IBC patients had tumor sizes ≤ 4 cm and 75% had sizes > 4 cm. Tumor grade analysis of non-IBC patients revealed that 12% had tumor grade I, 67% had tumor grade II and 20% tumor grade III. Tumor grade analysis of IBC patients revealed that 75% had tumor grade II and 21% tumor grade III. The status of lymph node metastasis showed that 61% of non-IBC had <4 and 39% had ≥4 metastatic lymph nodes. All IBC patients had positive lymph nodes metastasis with 25% <4 and 75% ≥4 metastatic lymph nodes. IBC patients possess significantly (*p* = 0.02) higher number of metastatic lymph nodes compared with non-IBC patients. Assessment of hormone and growth factor receptors ER, PR and HER-2 revealed that positivity was detected in 35%, 41% and 24% of the non-IBC patients respectively. For the IBC patients ER, PR and HER-2 receptor positivity was detected in 50%, 44% and 27%, respectively. Lymphovascular invasion was detected in 16% of non-IBC and 82% IBC patients respectively. Tumor emboli, a hallmark of IBC defined as clusters of tightly bound tumor cells retracted from the surrounding endothelial lining [32], [33], were detected in10% of non-IBC tissue sections but in all tissue sections of IBC (100%).Statistical analysis showed that tumor emboli are a highly dominant feature in IBC (*p* = 0.0001) compared with non-IBC carcinoma tissue.

**Table 1:** Clinical and pathological characterization of non-IBC versus IBC patients.

Characteristic	Non-IBC	IBC	*p* value
	(N = 49)	(N = 28)	
**Age**			
Rang	29–73	29–65	
Mean ±SD	53.3±11.3	48.9±9.9	0.4^a^
NA	1	0	
**Tumor size**			
Mean ±SD	4.5±3.4	5.8±2.4	
≤4	30(61%)	6(21%)	0.001* b
>4	17(34%)	21(75%)	
NA	2(5%)	1(4%)	
**Tumor grade**			
G1	6(12%)	0(0%)	
G2	33(67%)	21(75%)	0.1^b^
G3	10(20%)	6(21%)	
NA	0(0%)	1(4%)	
**Axillary Lymph Node Metastasis** †			
<4	30(61%)	7(25%)	0.02^b^
≥4	19(39%)	21(75%)	
**ER**			
Negative	27(65%)	14(50%)	0.8^b^
Positive	22 35%)	14(50%)	
**PR**			
Negative	29(59%)	13(46%)	0.3^b^
Positive	20(41%)	15(44%)	
**Her-2**			
Negative	37 (76%)	20(73%)	1.0^b^
Positive	12 (24%)	8(27%)	
**Lymphovascular invasion**			
Negative	40(82%)	5(18%)	
Positive	8(16%)	23(82%)	0.004* b
NA	1(2%)	0(0%)	
**Tumor emboli**			
Negative	44(90%)	0(0%)	0.0001* b
Positive	5(10%)	28(100%)	

Data are reported as means± SD

a Student’s t-test.

b Fisher’s exact test.

NA =  not available

* Significant p value calculated by:

† P value was calculated between <4 including 0 and ≥4.

### IBC Patients Characterized by Increase in HCMV IgG Antibody Titers

**Figure 1:**
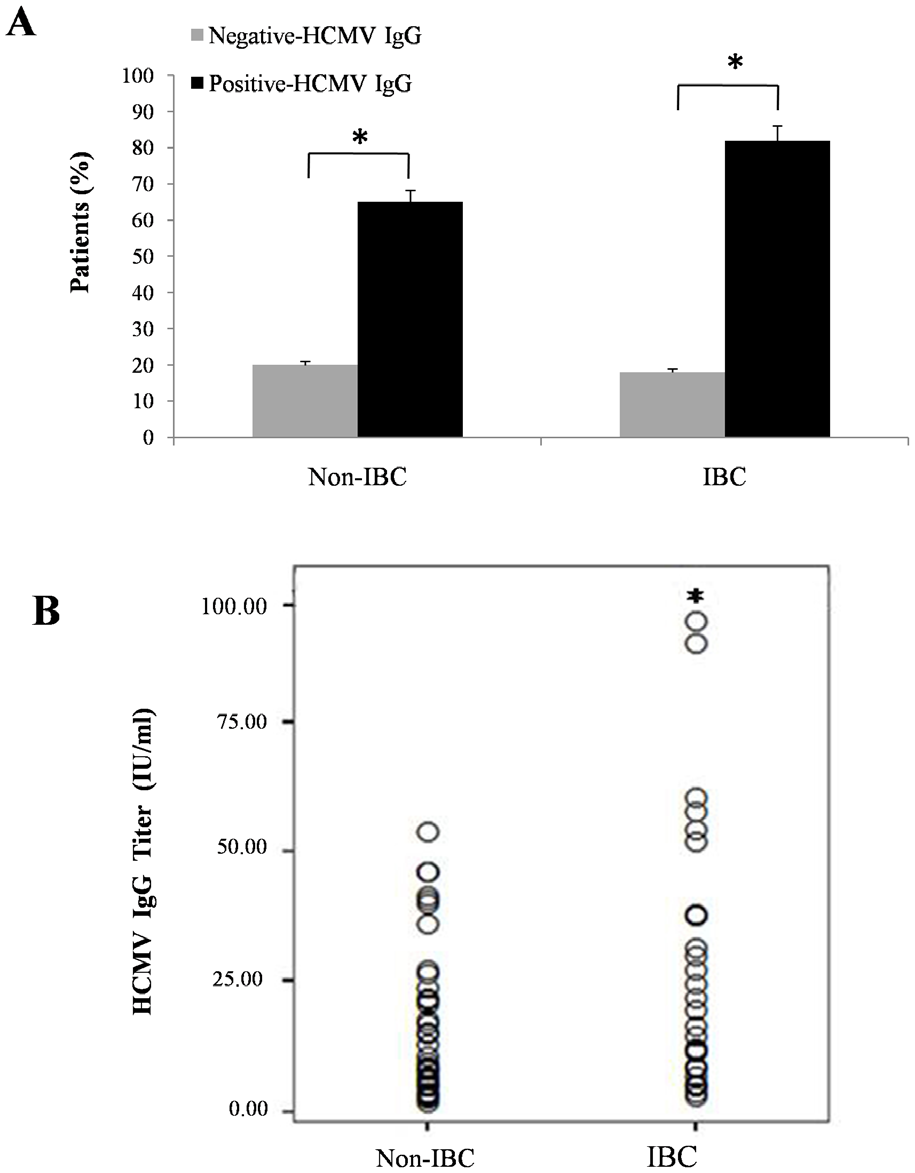
HCMV infection in non-IBC (n = 49) and IBC (n = 28) patients. (**A**) Bars represents percentage of serological diagnosis of HCMV-IgG within non-IBC (*p = *0.001) and IBC (*p* = 0.001) patient groups. *Indicates a significant *p* value as determined by Fisher’s exact test. (**B**) Serum titers of HCMV-IgG in non-IBC versus IBC in HCMV infected patients. Each dot represents one patient. Antibody titer of HCMV was significantly higher (*p* = 0.04) in IBC than in non-IBC. *Indicates a significant *p* value as determined by Student’s t-test.

HCMV specific IgG in the peripheral blood of breast cancer patients was detected in 65% of non-IBC patients (*p* = 0.001) and 82% of IBC patients (*p* = 0.001) (Fig. 1A). Our results are consistent with previous studies which found an association between HCMV IgG levels and breast cancer in young women [34]. However there was no difference in HCMV-IgG serological diagnosis between non-IBC and IBC patient groups. Comparing the mean value of HCMV IgG antibody titer in infected patients we found that in non-IBC patients (n = 42) the titer was 18.45±15.7 IU/mL, whereas in IBC patients (n = 28) it was 25.96±24.50 IU/mL. Statistical analysis revealed that IgG antibody titer was significantly higher (p=0.04) in IBC versus non-IBC patients (Fig. 1B).

### Significant Increase in the Incidence of HCMV-DNA in IBC Carcinoma Tissues

Standardized genomic DNA of peripheral blood, carcinoma and non-carcinoma tissues of non-IBC and IBC patients were subjected to nested PCR analysis for the fourth exon of the HCMV-IE gene as previously described [30]. De-identified positive control samples were serologically determined as positive for HCMV by IgG/IgM seropositive reaction and by real time PCR for HCMV-DNA using infected patient sera, obtained from the Ain Shams University laboratory. Negative controls were obtained from peripheral blood of healthy volunteers diagnosed as HCMV-seronegative.

We identified as positive HCMV-DNA when agarose gel electrophoresis of nested PCR products revealed a band corresponding to a 293-bp DNA fragment. Using nested PCR analysis we did not detect HCMV-DNA in peripheral blood of non-IBC and IBC patients (data not shown). In contrast analysis of carcinoma tissue showed that 53.1% of non-IBC carcinoma tissues were HCMV-DNA positive and 46.9% HCMV-DNA negative but in IBC patients 78.6% of carcinoma tissues were HCMV-DNA positive and 21.4% were HCMV-DNA negative (*p* = 0.030) (Table 2).

**Table 2:** Comparison of detected of HCMV-DNA in breast carcinoma tissues of non-IBC and IBC patients.

HCMV nested PCR	Non-IBC	IBC (%)	*p* value
	n(%)	n(%)	
Positive	26(53.1%)	22(78.58%)	0.030*
Negative	23(46.9%)	6(21.42%)	

Significant p value calculated Fisher’s exact test.

n: number of patients.

We tested HCMV-DNA in the non-carcinoma tissues of non-IBC and IBC patients, and in healthy breast tissue obtained at mammoplasty. Results showed that 16.66% of non-carcinoma tissues of non-IBC patients were HCMV-DNA positive and 83.33% HCMV-DNA negative. In contrast, all of the examined non-carcinoma tissues of IBC patients (100%) and healthy breast tissues (100%) were HCMV DNA negative (Fig. S1).

### Detection of Different HCMV Strains in IBC Versus Non-IBC Specimens

**Figure 2:**
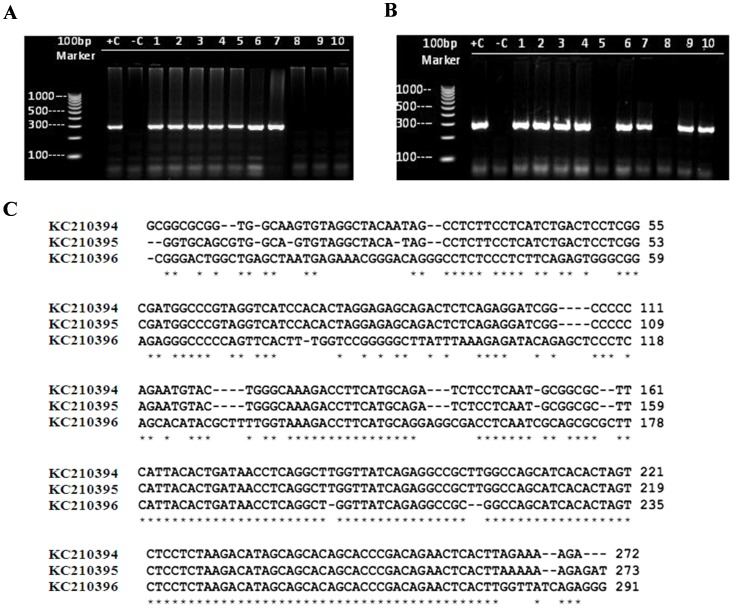
Agarose gel electrophoresis for nested PCR analysis of HCMV-DNA. Samples were considered positive when a band of 293bp was detected. (**A**) Representatives of non-IBC carcinoma tissue samples: lanes 1–7 display positive-HCMV; lanes 8–10 represent negative samples. (**B**) Representatives of IBC carcinoma tissue samples: lanes from 1–4, 6–7, 9–10 display positive HCMV; lanes 5 and 8 represent negative samples. Lanes +C represent HCMV positive control; lanes -C represent HCMV negative control. (**C**) Nucleotide sequence alignment from the purified nested PCR products. *represents nucleotide identity.

We sequenced purified PCR product of HCMV-DNA detected at 293-bp in control non-IBC (Fig. 2A) and IBC (Fig. 2B) carcinoma tissues compared to positive control. Nucleotide sequence analysis of the PCR product detected on positive control samples (Accession No: KC210393) showed (99%) identity with HCMV AD169 strain (Accession No: BK000394.5). Nucleotide sequence analysis of PCR product (n = 10) detected in non-IBC carcinoma tissues (Accession No: KC210395) showed a significant degree of identity (93–99%) with HCMV strain AL (Accession No: GQ222015.2). While nucleotide sequence analysis of PCR product detected in IBC carcinoma tissues revealed that 8 samples (Accession No: KC210395) had 99% identity to the HCMV AL strain (Accession number, GQ222015.2). One IBC sample (Accession No: KC210394) showed 99% identity with HCMV AD169 strain (Accession No: BK000394.5) and one IBC sample (Accession No: KC210396) showed (84%) identity to the HCMV NT strain (Accession No: GQ222016.2). The nucleotide sequence alignment of the identified HCMV PCR product is shown in (Fig. 2C). Polymorphisms are typically found among HCMV strains and may be associated with viral pathogenesis [35], [36].

### HCMV Infection Enhances Expression of NF-κB/p65 in IBC Carcinoma Tissue Samples

**Figure 3:**
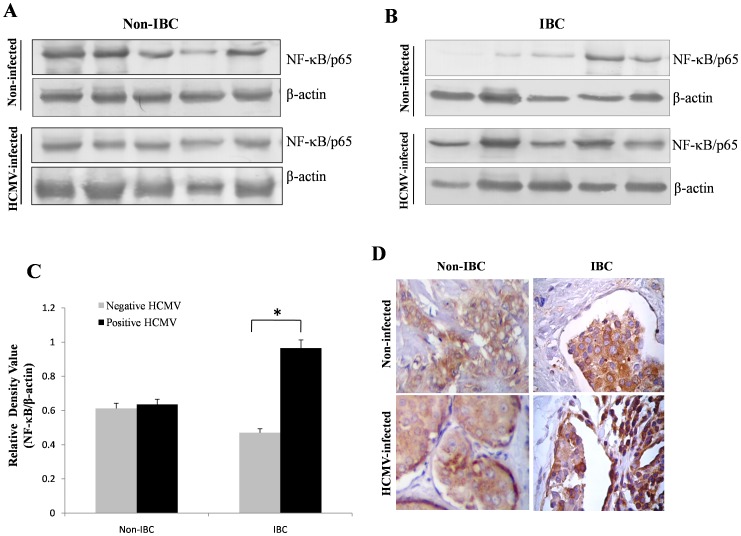
Expression of total NF-κB/p65 in randomly selected non-IBC and IBC cancer tissues. (**A**) Immunoblot analysis showing the expression of NF-κB/p65 (65 kDa) in tissue lysates of non-infected and HCMV-infected non-IBC patients; β-actin (45 kDa) was used as loading control. (**B**) Immunoblot analysis showing low level of expression of NF-κB/p65 in non-infected tissue lysates of IBC patients and high level of expression of NF-κB/p65 in the tissue lyastes of HCMV-infected IBC patients. Immunoblot results are representative of 3 independent experiments. (**C**) Bars represent relative densities of total NF-κB/p65 (mean ± S.D.) as quantified by imageJ software, showing significant increases in the level of expression of total NF-κB/p65 in tissue lysates of HCMV-infected IBC patients compared with tissue lysates of non-infected IBC patients. (**D**) Photomicrographs representative of immunohistochemistry staining of NF-κB/p65 (brown color) in non-IBC and IBC paraffin tissue sections with and without HCMV infection. Non-IBC intraductal carcinoma tissues showed moderate level of expression of NF-κB/p65 by non-infected and HCMV-infected tissues. On the other hand, NF-κB/p65 was moderately expressed by non-infected IBC tissue sections and over-expressed by IBC tissues infected with HCMV, (original magnification 400X). *Indicates significant *p* value as determined by Student’s t-test.

Previous studies showed that expression and up-regulation of the transcription factor NF-κB and its effector signaling proteins play a crucial role in IBC disease progression [37]–[39]. In addition, other studies reported that HCMV viral proteins interact with host cells and can up-regulate the expression of NF-κB signaling proteins [40], [41]. Building on these prior results, we hypothesized that up-regulation of NF-κB/p65 in IBC may be due in part to HCMV infection, either directly or indirectly as a result of the release of cytokines from infected immune cells. We therefore assessed the level of expression of total NF-κB/p65 in the infected and the non-infected carcinoma tissues of non-IBC and IBC patients. Using immunoblot analysis, we found that total NF-κB/p65 was expressed in the carcinoma tissue of both non-infected and HCMV-infected non-IBC patients (Fig. 3A). In IBC patients however, immunoblot analysis revealed that non-infected IBC carcinoma tissues showed lower expression of total NF-κB/p65 compared to HCMV-infected IBC carcinoma tissue (Fig. 3B). Densities of the immunoblot band of total NF-κB/p65 (65 kDa) were normalized to the level of the internal control β-actin (45 kDa) for each sample and the relative density values of NF-κB/p65 signals were quantified using imageJ software (Fig. 3C). Statistical analysis indicated that in the non-IBC carcinoma group there was no difference in the level of expression of total NF-κB/p65 between non-infected and HCMV-infected carcinoma tissues but in HCMV-infected IBC carcinoma tissue total NF-κB/p65 was significantly higher (*p* = 0.00023) than in non-infected carcinoma tissue.

Tissue localization of total NF-κB/p65 was assessed in paraffin sections using immunohistochemistry (Fig. 3D). In the non-IBC carcinoma tissues, the non-infected and HCMV-infected tissue sections showed equal levels of expression of total NF-κB/p65 detected by moderate intensity of the NF-κB/p65 brown stain color (score 2). In the IBC tissue sections, the non-infected carcinoma tissues showed moderate expression (score 2) of NF-κB/p65, while HCMV-infected tissues, showed strong expression of the transcription factor NF-κB/p65 (score 3).

**Figure 4:**
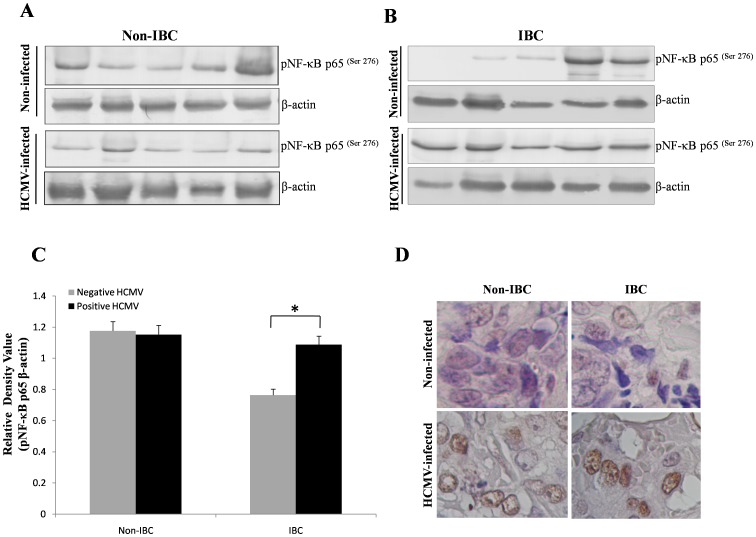
Expression of phospho-NF-κB p65 (pNF-κB p65 ^(Ser 276)^) in randomly selected non-IBC and IBC cancer tissue. (**A**) Immunoblot analysis showing equal level of expression of pNF-κB p65 ^(Ser 276)^ (65 kDa) in tissue lysates of non-infected and HCMV-infected non-IBC patients; β-actin (45 kDa) was used as loading control. (**B**) Immunoblot analysis showing low expression level of phospho-NF-κB p65 (Ser^276^) in the non-infected tissue lysates of IBC patients and greater of expression of pNF-κB p65 ^(Ser 276)^ in the tissue lyastes of HCMV-infected IBC patients. Immunoblot results are representative of at least 3 independent experiments. (**C**) Bars represent relative densities (mean ± S.D.) of pNF-κB p65 ^(Ser 276)^ as quantified by imageJ software. Statistical analysis revealed no significant differences in the expression of pNF-κB p65 ^(Ser 276)^ in tissue lysates of non-infected compared with HCMV-infected patients in non-IBC and IBC patient groups. (**D**) Photomicrographs are representative of immunohistochemical staining of pNF-κB p65 ^(Ser 276)^ in non-IBC and IBC paraffin tissue sections of patients not infected and infected with HCMV. There was low expression of pNF-κB p65 ^(Ser 276)^ in non-infected tissues of non-IBC and IBC. But pNF-κB p65 ^(Ser 276)^ was moderately expressed in non-IBC and IBC HCMV-infected patients (original magnifications 400X). *Indicates significant *p* value as determined by Student’s t-test.

To test whether HCMV infection is associated with activation/phosphorylation of NF-κB/p65 (Ser^276^), we measured phospho-NF-kB p65 (Ser^276^) in HCMV infected and non-infected carcinoma tissues of non-IBC and IBC patients using immunoblot and immunohistochemistry. Immunoblot analysis revealed that phospho-NF-κB p65 (Ser^276^) was detected in the non-infected and HCMV-infected carcinoma tissues of non-IBC patients (Fig. 4A). However in IBC patients phospho-NF-κB p65 (Ser^276^) was less expressed in the non-infected carcinoma tissue compared with HCMV-infected carcinoma tissue (Fig. 4B). The relative densities of phospho-NF-kB p65 band signals were measured using ImageJ software and there was no difference in the level of expression of phospho-NF-κB p65 between the non-infected and HCMV-infected carcinoma tissues of non-IBC patients. On the other hand, in IBC patients there was a significant increase (*p* = 0.048) in the level of expression of phospho-NF-κB p65 (Ser^276^) in infected versus non-infected IBC carcinoma tissue (Fig. 4C ). Thus, HCMV infection correlated with activation of NF-κB p65 in IBC patients.

Immunohistochemical localization of phospho-NF-κB p65 (Ser^276^) was assessed using immunohistochemistry (Fig. 4D). In non-IBC patients nuclear phospho-NF-κB p65 (Ser^276^) was weakly detectable in the non-infected tissues (score ranges from 0–1) compared with HCMV-infected tissues which showed weak to moderate intensity of phospho-NF-κB p65 staining (score ranges 1–2). In IBC patients, non-infected carcinoma tissues showed weak expression of phospho-NF-κB p65 (scores from 0–1) compared with moderate expression of NF-κB/p65 (scores 1–2) in HCMV-infected tissues.

## Discussion

Investigating the role of HCMV in cancer etiology was recommended following the development of advanced and sensitive laboratory techniques that can detect virus genomic, protein and secretome products in cancer tissues [42]. Studies have shown that HCMV-DNA can be detected in colorectal cancer tissues but not in normal tissues of the colon [7], and in carcinoma tissue of malignant glioma [15] and prostate cancer [8]. Moreover, HCMV infection was implicated as a key determinant of clinical outcome in glioblastoma. For example, patients with HCMV microinfection were found to have higher survival rates compared with patients with detectably higher levels of HCMV infection in cancer tissue [43].

High levels of antibody titers to herpes viruses have been previously detected in human cancers, including HCMV in newly diagnosed [44] and metastatic breast cancer patients [45]. Furthermore, HCMV proteins and viral DNA were detected in breast ductal carcinoma in situ and infiltrating ductal carcinomas, suggesting a correlation if not a role for HCMV in breast cancer [10]. That said, the effect off HCMV infection on breast cancer development and progression has been poorly investigated at best.

IBC is considered to be a unique phenotype of breast cancer characterized by rapid dissemination, poor prognosis and low survival rate [46]. Moreover, the biological mechanisms underlying IBC disease biology are poorly understood. Our studies show that IBC tissues are characterized by over expression of proteases such as cysteine protease cathepsin B [29], membrane type matrix metalloproteinase-1 (MT1-MMP) and matrix metalloproteinase-2 and -9 (MMP-2 and MMP-9) [28]. Infection of HCMV was found to induce the expression of different cytokines and chemokines among which were TNF-α, IL10, IL-8 and MCP-1 [47], [48]. Furthermore, studies suggested that HCMV infection induces overexpression of MMPs in mesenchymally transformed renal tubular cells [49]. Therefore, based on published results and other studies cited above, we suggest that HCMV may be involved in the etiology and progression of IBC.

In the present study we screened for HCMV infection in non-IBC and IBC patients. Serological diagnosis indicated that the HCMV antibody titer was higher in IBC patients than in non-IBC cases. In addition, a high antibody titer for HCMV has been detected in patients newly diagnosed with breast cancer [44]. Furthermore, there was a significant difference in HCMV-DNA detected in carcinoma tissue of IBC compared with versus non-IBC patients, suggesting a possible role for HCMV in the pathobiology of IBC; this role now needs to be examined closely to assess possible causality. Data that are most compelling for an association between HCMV and IBC are sequence analysis data of the detected HCMV-DNA, for which our findings show that HCMV infected IBC tissues possess multiple and different strains of viral DNA than that observed in non-IBC breast cancer tissues.

IBC was associated with HCMV strains AL, NT and AD169, whereas in infected non-IBC tissues we only found HCMV strain AL and at a considerably lower frequency. In our positive control HCMV infected patients that did not have breast cancer we found only HCMV strain AD169. There is a need to explore polymorphism differences among HCMV strains which may provide additional information possibly implicating involvement of HCMV in IBC disease etiology.

HCMV infection was previously shown to augment angiogenesis and lymphangiogenesis [21], to induce cancer cell motility, invasion and adhesion to endothelial cells [50], [51], to stimulate NF-κB signaling pathways [40] and to promote resistance of cancer cells to chemotherapy [52], all of which are also properties that characterize IBC disease [33], [39], [53], [54]. Thus, we suggest that HCMV may contribute to the specific phenotype of IBC, either directly or indirectly, by activating specific cellular pathways such as that of transcription factor NF-*κ*B, which itself is associated with poor prognosis in breast cancer. Molecular studies indicate that genes activated by the NF-*κ*B signaling pathway are over-expressed and activated in IBC versus non-IBC [37]–[39]. Now having shown that NF-*κ*B is strongly activated in IBC, it is very likely that its activation and sustained high activity contribute to IBC disease aggressiveness. Most interestingly, when we tested the level of expression and cellular localization of total and phospho-NF-*κ*B/p65 in HCMV-infected and non-infected tissues of IBC and non-IBC patients, we found a significant increase in the expression and activity of NF-*κ*B/p65 in HCMV-infected IBC carcinoma tissue compared with non-infected carcinoma tissue. In fact, in the non-IBC breast carcinoma tissue samples there was no difference in the level of expression or activity of NF-*κ*B/p65 in the non-infected compared with infected carcinoma tissue. Increased expression and activation of NF-*κ*B molecules following HCMV infection have been found to be regulated by different mechanisms, including transactivation by HCMV IE genes such as IE1-72, IE2-86 and IE2-55, and increased DNA binding activity of transcription factor SP1 [40].

A significant increase in the expression of NF-*κ*B/p65 in infected IBC carcinoma tissue may be due to the secondary involvement of biological molecules such as cytokines and chemokines that characterize IBC tumor biology, but which could be elevated due to HCMV infection of immune cells, fibroblasts or epithelial cells, and in which HCMV induces oncomodulatory proteins through activating NF-*κ*B/p65 signaling pathways [55]. Currently, we are establishing *in-vitro* tissue culture models with and without HCMV infection to identify candidate molecules that result from HCMV infection, an area that has received very little attention.

In conclusion, our results suggest that HCMV infection correlates with IBC pathogenic phenotype and may contributes to the etiology of IBC, directly or indirectly. If this is true additional therapeutic targeting of HCMV may be warranted in the treatment of IBC. It will be important to determine whether HCMV infection either enhances disease severity or whether IBC disease is more susceptible to HCMV infection.

## Supporting Information

Figure S1 **Agarose gel electrophoresis for nested PCR analysis of HCMV DNA detected in mammoplasty, non-carinoma and carcinoma tissues of non-IBC and IBC patients.** (**A)** Representatives of the non-carcinoma and carcinoma tissue samples of non-IBC patient: C− represents negative control, C+ represents positive control, M represents mammoplasty breast tissue, (1N, 2N, 3N and 4N) represents non-cancer tissue of patients sample (1CT, 2CT, 3CT and 4CT) represents cancer tissues of patients samples. (**B)** Representatives of non-carcinoma and carcinoma tissue samples of IBC patinets: C− represents negative control, C+ represents positive control, M represents mammoplasty breast tissue, (1N, 2N, 3N and 4N) represents non-cancer tissue of patients sample (1CT, 2CT, 3CT and 4CT) represents cancer tissues of patients samples. (**C**) Bars represents percentage of HCMV infected tissues within mammoplasty control, non-IBC, and IBC tissues groups. HCMV DNA was not detected in mammoplasty tissues of healthy volunteers. In non-IBC patients HCMV DNA was detected in 83.33% of cancer tissues and in 16.66% (2 patients samples out of 49) non-cancer tissues. In IBC patients HCMV DNA was detected in 100% of cancer tissues and was not detected in non-cancer tissues. *Indicates significant *p* value as determined by Fisher’s exact test.
(TIF)
