# Supplementary figures and images for: Human Cytomegalovirus Infection Enhances NF-κB/p65 Signaling in Inflammatory Breast Cancer Patients
Source: PLoS One. 2013 Feb 13;8(2):e55755. doi: 10.1371/journal.pone.0055755 (PMC3572094; doi:10.1371/journal.pone.0055755)

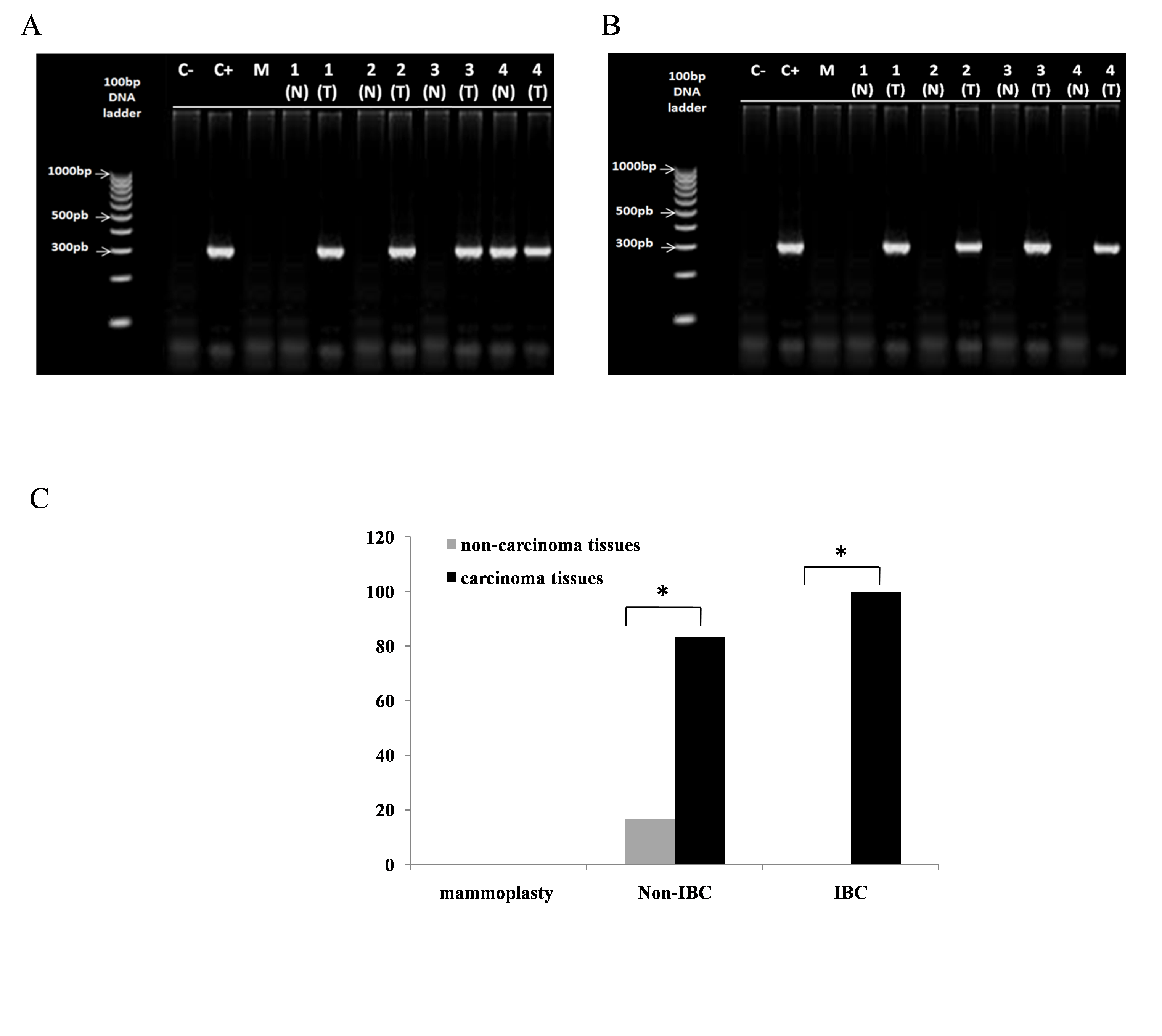

Supplement: Figure S1 — Agarose gel electrophoresis for nested PCR analysis of HCMV DNA detected in mammoplasty, non-carinoma and carcinoma tissues of non-IBC and IBC patients. (A) Representatives of the non-carcinoma and carcinoma tissue samples of non-IBC patient: C− represents negative control, C+ represents positive control, M represents mammoplasty breast tissue, (1N, 2N, 3N and 4N) represents non-cancer tissue of patients sample (1CT, 2CT, 3CT and 4CT) represents cancer tissues of patients samples. (B) Representatives of non-carcinoma and carcinoma tissue samples of IBC patinets: C− represents negative control, C+ represents positive control, M represents mammoplasty breast tissue, (1N, 2N, 3N and 4N) represents non-cancer tissue of patients sample (1CT, 2CT, 3CT and 4CT) represents cancer tissues of patients samples. (C) Bars represents percentage of HCMV infected tissues within mammoplasty control, non-IBC, and IBC tissues groups. HCMV DNA was not detected in mammoplasty tissues of healthy volunteers. In non-IBC patients HCMV DNA was detected in 83.33% of cancer tissues and in 16.66% (2 patients samples out of 49) non-cancer tissues. In IBC patients HCMV DNA was detected in 100% of cancer tissues and was not detected in non-cancer tissues. *Indicates significant p value as determined by Fisher’s exact test. (TIF) [file pone.0055755.s001.tif]
